# Comment on “Scar‐Degrading Endothelial Cells as a Treatment for Advanced Liver Fibrosis”

**DOI:** 10.1002/advs.202207396

**Published:** 2023-03-18

**Authors:** David M. Dolivo, Adrian E. Rodrigues, Thomas A. Mustoe, Robert D. Galiano, Seok Jong Hong

**Affiliations:** ^1^ Division of Plastic Surgery Department of Surgery Northwestern University‐Feinberg School of Medicine Chicago IL 60611 USA

**Keywords:** extracellular matrix, fibrosis, scar

## Abstract

Cellular therapies show promise for treatment of fibrosis. A recent article presents a strategy and proof‐of‐concept for delivering stimulated cells to degrade hepatic collagen in vivo. A discussion is presented surrounding the strengths of this approach and the potential to generalize this strategy of optimizing cell sources and activation stimuli to treat other types of fibrosis.

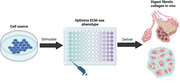

We recently read Zhao et al.^[^ ^1^ ^]^ with great interest. In this report, the authors describe a unique and intriguing method to screen for cells with substantial collagenase activity, followed by extensive characterization of these cells’ ability to degrade fibrotic extracellular matrix (ECM) in a murine model in vivo and in human samples ex vivo. We believe that the authors should be commended for the novelty and depth of the work presented here, and we are particularly excited about the potential for translation of this approach to other types of fibrosis.

Fibrosis is a nonregenerative repair process by which damaged, formerly functional tissue is replaced with a collagenous, mechanically aberrant scar. As a pathophysiological outcome of myriad disease states of multiple tissues, fibrosis can lead to damage and loss‐of‐function in numerous organs including the liver, skin, lung, kidney, and heart. Due to the variety of clinical conditions that can manifest as development of tissue fibrosis, frequently cited statistics estimate that nearly half of deaths in the industrialized world may be attributed to fibrosis.^[^ ^2^ ^]^ Therefore, the need for therapeutic modalities to prevent and treat fibrosis is paramount, though success in clinical translation of antifibrotic pharmacologic therapies has been disappointing. Another potential treatment strategy is to deliver therapeutic cells, but this is complicated by the challenge of selecting appropriate cells with the potential to prevent or reverse fibrosis, as well as to deliver these cells effectively and efficiently to the tissue of interest.

In Zhao et al.,^[^ ^1^ ^]^ the authors aimed to select a source of cells with a high degree of collagenase activity to be used as a cellular therapy to degrade liver collagen in an advanced stage hepatic fibrosis model in vivo. The authors initially performed a high‐throughput screen for collagenase activity by seeding various preparations of cells into a collagen matrix pretagged with rhodamine, such that fluorescence of the culture supernatant could be used as a proxy for collagenase activity. In their initial screen, the authors found notably elevated collagenase activity in human liver sinusoidal endothelial cells treated with accutase and phorbol‐12‐myristate‐13‐acetate, which was particularly appropriate given their subsequent focus on hepatic fibrosis. However, there is nothing inherent to this approach that is limited to this cell source or to this set of stimuli. Studies of endogenous physical and biochemical stimuli regulating proteases, including collagenases, have been published in the literature for decades vis‐à‐vis investigation of “normal” and dysregulated wound healing;^[^ ^3^, ^4^, ^5^, ^6^ ^]^ such stimuli might be explored in order to expand upon the list of compounds assayed in the authors’ screen. Furthermore, with the wealth of “‐omic” data now available in reference to fibrotic disease states both in animal models and in human patients,^[^ ^7^, ^8^ ^]^ we continue to better understand which gene expression paradigms and extracellular matrix profiles are shared among disease states, and which are characteristic of specific fibrotic pathologies. Other types of cells may be investigated as well. A wealth of publicly available gene expression data enables in silico prediction of cell types that may have heightened or suppressed activity to degrade particular ECM components, based on expression of genes encoding proteases and protease inhibitors of known target specificities.^[^ ^9^, ^10^ ^]^ Thus, tissue‐specific fibrotic ECM data and cell type‐specific transcriptomic profiles could be used to further individualize ECM‐ase screens to identify cell‐stimulus combinations that are uniquely well‐suited to degrade particular configurations of fibrotic ECM. These approaches will only become more powerful as more data are generated and become widely available.

Intriguingly, authors demonstrated that delivery of ECM‐degrading liver sinusoidal endothelial cells (dLSECs) via intrasplenic or intraperitoneal injection resulted in functional collagenase activity and subsequent reduction of collagen content in experimental late‐stage murine hepatic fibrosis. This lies in contrast to much of the experimental literature, which evaluates therapeutic modalities for their ability to prevent or antagonize early signals that trigger the beginning of fibrosis. While preventing the initial development of fibrosis is arguably a more facile and straightforward scientific problem to address, compared to treatment of existing fibrosis, it has only limited relevance to common clinical scenarios, in which patients frequently present with advanced tissue fibrosis.^[^ ^11^ ^]^ Notably, Zhao et al. demonstrate that, 5 days after administration of dLSECs, no significant liver regeneration has occurred as assessed by transcript expression and immunohistochemical markers. Though this may seem disappointing at first, the timepoint of 5 days postdelivery is still very early, particularly considering that these mice had already been subjected to 15 prior weeks of carbon tetrachloride injections. We maintain that assessment of functional liver regeneration after dLSEC therapy would be far more meaningful at later time points, after endogenous liver cells have had a chance to sense and respond to their new physiochemical and biomechanical microenvironment by activating pro‐regenerative epigenetic and transcriptional programs. Further, the authors demonstrated that, even 5 days after a single injection, some dLSECs still remained in the mouse liver, raising the possibility that the collagenase potential of their cellular therapy had not yet been exhausted at this time. Follow‐up at further time points will be critical not only to determine the eventual fate of the remaining cells, but also to assess hepatic regeneration over time. This should be investigated not solely by histological analysis, but also by quantification of typical, clinically relevant liver function blood test parameters like alanine transaminase, aspartate transaminase, and alkaline phosphatase, in order to monitor any recovery in hepatic function.

It may be that the liver is particularly suited for collagen‐degrading therapy in late‐stage disease since hepatic tissue, even in humans, contains remarkable propensity for regeneration that is lacking in other organs.^[^ ^12^, ^13^ ^]^ For some fibrotic conditions, such as dermal scarring after skin excision or myocardial scarring forming in response to infarction, fibrosis plays a critical role in re‐establishing tissue boundaries that have been lost, in order to maintain tissue continuity and preserve organ functional capacity. After all, scarred skin, though lacking in many ways, still provides a better physical and biochemical barrier to the outside world than an open wound. Therefore, it remains to be seen how degradation of collagen that has already formed and integrated into a fibrotic tissue will affect that tissue, and whether loss of fibrotic collagen will be accompanied by gradual structural and functional regeneration in the tissue of interest, even in organs that lack the prodigious regenerative properties of the liver. This provides a fascinating possibility with many open questions that we look forward to helping find the answers to, and we praise the excellent work of Zhao et al. for drawing attention to these intriguing ideas.

## Conflict of Interest

The authors declare no conflict of interest.
